# Deshima 2.0: Rapid Redshift Surveys and Multi-line Spectroscopy of Dusty Galaxies

**DOI:** 10.1007/s10909-022-02730-y

**Published:** 2022-05-05

**Authors:** M. Rybak, T. Bakx, J. Baselmans, K. Karatsu, K. Kohno, T. Takekoshi, Y. Tamura, A. Taniguchi, P. van der Werf, A. Endo

**Affiliations:** 1 Faculty of Electrical Engineering, Mathematics and Computer Science, Delft University of Technology, Mekelweg 4, 2628 CD Delft, The Netherlands; 2 Leiden Observatory, Leiden University, Niels Bohrweg 2, 2333 CA Leiden, The Netherlands; 3 Division of Particle and Astrophysical Science, Graduate School of Science, Nagoya University, Furocho, Chikusa-ku, Nagoya, Aichi 464-8602 Japan; 4 National Astronomical Observatory of Japan, 2-21-1 Osawa, Mitaka, Tokyo 181-8588 Japan; 5 SRON – Netherlands Institute for Space Research, Niels Bohrweg 4, 2333 CA Leiden, The Netherlands; 6 Institute of Astronomy, Graduate School of Science, The University of Tokyo, 2-21-1 Osawa, Mitaka, Tokyo 181-0015 Japan; 7 Research Center for the Early Universe, Graduate School of Science, The University of Tokyo, 7-3-1 Hongo, Bunkyo-ku, Tokyo 113-0033 Japan; 8 Kitami Institute of Technology, 165 Koen-cho, Kitami, Hokkaido 090-8507 Japan

**Keywords:** (sub)mm astronomy, Spectroscopy, High-redshift universe, Galaxies, Integrated superconducting spectrometer

## Abstract

We present a feasibility study for the high-redshift galaxy part of the Science Verification Campaign with the 220–440 GHz deshima 2.0 integrated superconducting spectrometer on the ASTE telescope. The first version of the deshima 2.0 chip has been recently manufactured and tested in the lab. Based on these realistic performance measurements, we evaluate potential target samples and prospects for detecting the [CII] and CO emission lines. The planned observations comprise two distinct, but complementary objectives: (1) acquiring spectroscopic redshifts for dusty galaxies selected in far-infrared/mm-wave surveys; (2) multi-line observations to infer physical conditions in dusty galaxies.

## Introduction

Throughout cosmic history, more than half of all the stars form in dust-obscured galaxies [1–3]. Due to their massive dust reservoirs, these dusty star-forming galaxies (DSFGs) are often invisible in the optical/near-IR part of the spectrum but bright in the far-infrared (FIR) to (sub)-mm wavelengths. Consequently, thousands of DSFGs have been identified in wide-field continuum surveys in the 0.1–2.0 mm regime [2–4].

However, studies of DSFGs suffer from a considerable *redshift bottleneck*. This is because sub-mm continuum observations probe the Rayleigh-Jeans tail of the dust thermal emission (which peaks at $\ge$1 THz rest-frame) and thus provide only weak constraints on the redshift. Moreover, the optical and near-IR spectroscopy is often inefficient due to the high extinction in DSFGs, particularly in the FIR-brightest sources [2]. However, robust redshifts are a prerequisite for emission line follow-up with interferometers such as ALMA [5].

Consequently, (sub)mm-wave spectroscopy has become the key to obtaining robust spectroscopic redshifts for dusty galaxies at high redshift. This is chiefly through the rotational emission line of ^12^CO and the fine-structure transition of C^+^, the 158-μm [CII] line (rest-frame frequency $f_0 = 1900.5$ GHz). Typically, these are conducted using heterodyne receivers via spectral scans, requiring multiple instrument tunings.

Alternatively, several dedicated wide-band instruments have been developed: e.g., grating spectrometers such as the now-defunct Z-Spec [6, 7] at the Caltech Submilimeter Observatory (CSO; 190–305 GHz) or wideband heterodyne receivers such as the ZSpectrometer [8] at the Green Bank Telescope (26.5-40 GHz) and the Redshift Search Receiver (RSR [9], 73–111 GHz) on the Large Millimeter Telescope. While these have allowed redshift measurements out to $z\simeq 6$ [10], mainly using the CO emission lines, due to the relative faintness of these lines, such observations are limited to the rare, very bright galaxies. Critically, the bright [CII] fine-structure line—ideal for rapid redshift measurements due to its brightness—is generally beyond the reach of these instruments.

To properly exploit the [CII] line for redshift measurements, wideband spectroscopy must be extended to higher frequencies, i.e. the 350-GHz and 400-GHz atmospheric windows. These frequency bands are particularly promising for spectroscopic confirmation of DSFGs, because the number density of DSFGs (and thus [CII] emitters) peaks between $z=2-4$ [11], corresponding to [CII] being redshifted to 380–600 GHz.

Here, we demonstrate the performance of deshima (DEep Spectroscopic HIgh-redshift MApper), a mm-wave integrated superconducting spectrometer (ISS) [12, 13], which combines superconducting filterbank with an array of microwave kinetic inductance detectors (MKIDs) on a single chip. Several other MKID spectrometers are currently under development, e.g., CONCERTO [14, 15] (130–310 GHz), and Super-Spec [16, 17] (190–310 GHz). deshima’s octave-wide bandwidth and high-frequency capability make it ideally suited for rapid redshift measurements.

## Deshima 2.0: Instrument Description

The deshima 1.0 prototype achieved the first light in 2017 [13, 18]. deshima offered an instantaneous bandwidth of 335–377 GHz with $R\sim 380$. These observations demonstrated the spectroscopy of point sources (post-AGB star IRC+10216 and a merging galaxy pair VV 114) and spectroscopic mapping of extended regions (the Orion KL star-forming region and a nearby galaxy NGC 253). However, the limited chip coupling efficiency ($\sim 2$%) and large overheads did not allow science-grade observations of high-z DSFGs).

In 2022, an upgraded deshima 2.0 spectrometer will be installed at the ASTE (Atacama Submillimeter Telescope Experiment) 10-meter telescope [19] in the Atacama desert, Chile, at an altitude of 4860 metres. deshima 2.0 will provide an instantaneous frequency coverage of 220–440 GHz at $R\simeq 500$ ($\Delta v \simeq$ 600 km/s). Besides the significantly expanded bandwidth, major upgrades between deshima 1.0 and deshima 2.0 include a leaky-lens antenna [20], improved filter design, and a sky-position chopper^1^. Together, these upgrades result in a factor 4–8 improvement in sensitivity over deshima 1.0. Further sensitivity improvements could be achieved by explicitly modelling the instrument and atmospheric noise, rather than simply subtracting the on- and off-source spectra [22].

The first version of the deshima 2.0 on-chip filterbank has been recently manufactured and tested in the lab [21]. The filters cover almost the entire target bandwidth, with a mean peak coupling efficiency of 14%, increasing up to 30–50% for some channels (target: $\sim$30%). The main source of discrepancy between the current and target sensitivity is uneven channel spacing, which reduces the coupling efficiency of individual channels.

**Fig. 1:**
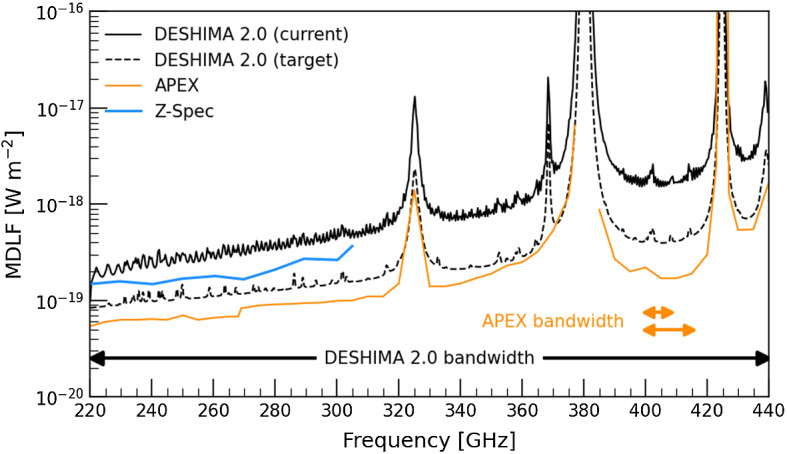
5$\sigma$ minimum detectable line flux (MDLF) for a 600 km/s wide line for the current and target deshima 2.0 sensitivity [21], compared to the Z-Spec [23] and the current suite of APEX receivers. For all instruments, we assume PWV = 0.6 mm, target elevation of 60 deg and on-source time of 3.6 hr per tuning. The instantaneous bandwidth of deshima 2.0 corresponds to 20 APEX tunings (APEX bandwidth = 8.0–15.8 GHz, depending on the receiver) (Color figure online)

In Fig. 1, we show the current and target deshima 2.0 sensitivity compared to Z-Spec [23] at the CSO on Mauna Kea, and the current suite of receivers on the 12-m Atacama Pathfinder EXperiment (APEX) telescope^2^. Compared to the Z-Spec, deshima 2.0 has is 1.5–2.0$\times$ less sensitive at a given precipitable water vapor (PWV) value. However, ASTE has more favourable weather conditions (PWV = 0.6 mm corresponds to the top 25th annual percentile for ASTE, but only the 10th percentile for Mauna Kea [24]). deshima 2.0 is thus competitive with Z-Spec, with the added advantage of covering the 305-440 GHz range.

Compared to APEX, deshima 2.0 is currently 4–5$\times$ less sensitive; further improvements might reduce this dicrepancy by a factor of 2. At that point, science goals that would require four or more APEX tunings will be more economically achieved with deshima 2.0. For such applications, deshima 2.0 will be directly competitive with APEX.

## Deshima 2.0: Science Verification Campaign Targets

The primary targets for deshima are DSFGs with large apparent FIR luminosity (i.e. $L_\mathrm {8-1100\mu m}$ of few $\times 10^{13} L_\odot$), many of which are strongly gravitationally lensed. In fact, hundreds of strongly lensed dusty galaxies were discovered in wide-field continuum surveys at FIR and sub-mm wavelengths with *Herschel* [25–27] and *Planck* satellites [28] and the South Pole Telescope [29] (SPT).

**Fig. 2:**
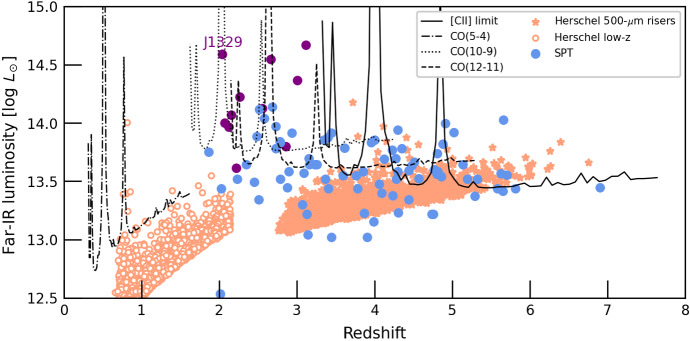
Distribution of redshift and apparent FIR luminosity of bright DSFGs from the SPT, *Planck* and the *Herschel* surveys, compared to the current deshima 2.0 sensitivity. The black lines indicate the 5$\sigma$ detection limits for the CO(5–4), (10–9), (12–11), and [CII] emission from individual sources, assuming empirical CO-FIR ratios [30, 31] and a [CII]/FIR ratio of $10^{-3}$. We assume 12-hr observations at PWV = 1.0 mm, i.e. less than two median observing nights. Galaxies at $z\le 3.3$ have the [CII] line outside deshima 2.0 frequency coverage, but will be prime targets for multi-line spectroscopy. We highlight J1329+2243—a very bright $z=2.05$ lensed DSFGs, a simulated spectrum for which is presented in Fig. 3 (Color figure online)

Fig. 2 shows the redshift and apparent FIR luminosity distribution of DSFGs from the SPT and *Planck* samples (virtually all with spectroscopic redshifts) and the *Herschel*-selected high/low-redshift samples. For comparison, we show the limiting FIR luminosity of sources for which the CO (5–4), (10–9), (13–12) and the [CII] line can be detected at 5$\sigma$ level in 5-hr on source ($\sim$12 hr total with overheads). These are based on empirical CO–FIR relations from Kamenetzky et al. [30] for CO (5–4), Greve et al. [31] for CO (10–9) and (13–12), and $L_\mathrm {[CII]}/L_\mathrm {FIR}$ ratio of $10^{-3}$, typical for DSFGs [32, 33]. The CO (and [CII]) luminosities in individual galaxies can deviate from these trends by up to 1 dex [30, 31, 34, 35]; large-sample surveys with deshima 2.0 will further constrain the range of CO excitation in DSFGs.

### Spectroscopic Redshifts for Bright *Herschel*-Selected Galaxies

One of the primary aims of the deshima 2.0 Science Verification Campaign is to demonstrate the rapid redshift acquisition capability. The atmospheric windows and bandwidth of deshima offer two promising regions for efficient redshift searches.

Firstly, at lower-redshift ($z \sim 0.5 - 2.0$), the large bandwidth of deshima offers multiple-line detections of galaxies. Counterintuitively, finding robust redshifts of lower-redshift dust-obscured galaxies is challenging. While the spectral lines suffer less from the cosmological dimming, wider bandwidths are necessary to cover the entire possible redshift space. In addition, the SPT and *Planck* surveys select DSFGs at $z\gtrsim 2$, while *Herschel* redshift follow-up prioritises high-*z* targets. The *Herschel* catalogues have thus left $\sim 4000$ $z \sim 1$ targets unexplored. As $z\le 1$ DSFGs have low lensing probability [36], this sample presents a population of intrinsically-bright galaxies after the peak of the cosmic star-forming activity [1], i.e. when galaxy-wide quenching should be in full effect. deshima’s 220-GHz bandwidth will allow fast redshift acquisition for these sources, removing degeneracies in redshift due to the wide spacing of the CO lines at low redshift [37].

As for the high-redshift end, while most bright DSFGs have secure spectroscopic redshifts, a large population of DSFGs with lower apparent luminosities remains unexplored. Namely, the $\ge$1000 deg$^2$ *Herschel* footprint [38, 39] provides a sample of $\approx$ 2000 “500-$\mu$m risers”: DSFGs with flux density peaking at/beyond 500-$\mu$m. A 500-$\mu$m rising colour selection, relative to 250 and 350 $\mu$m, promises to select the highest-redshift *Herschel* candidates [40], with $z_\mathrm{phot} \ge 3.5$. A major advantage of deshima 2.0 is the wideband spectroscopy in the 385-440 GHz band (interrupted by the 425-GHz telluric line), corresponding to the lower half of ALMA Band 8. This enables [CII] observations at $z=3.3-3.9$, the epoch when the [CII] luminosity function is predicted to peak [11].

We expect to invest a total of 400 hr for both the low- and high-redshift goal (200 hr per goal); this should yield robust redshifts for $\sim 15 - 20$ galaxies each.

**Fig. 3:**
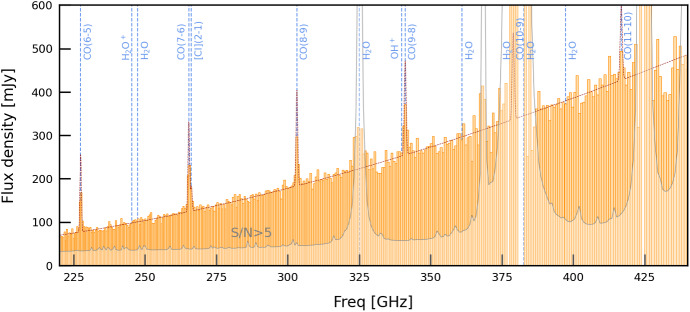
A simulated deshima 2.0 observation of J1329+2243, assuming 3 hours on-source at 40 deg elevation and PWV = 0.6 mm. The orange bars show the response for each channel. The red line shows the input spectrum based on existing observations [35]. The grey line denotes the S/N = 5 threshold for each channel; increasing the on-source time will move the grey line downwards. We expect to detect four CO rotational lines; our observations will also additional spectral lines—a key discovery space for deshima 2.0 (Color figure online)

### Multi-line Spectroscopy of Bright Lensed DSFGs

In addition to measuring redshifts, mm-wave spectroscopy provides critical insights into the physical conditions in DSFGs. Namely, observations of multiple chemical species (e.g., CO, C$^+$, C, O, dust continuum) can be linked to the underlying physical conditions (e.g., gas density and temperature, irradiation, turbulence) using chemical and radiative transfer modelling. For example, the [CII] 158-$\mu$m line is a sensitive probe of the far-UV irradiation. The excitation of CO rotational lines is primarily driven by gas density [41, 42], but the $J_\mathrm {upp}\ge 8$ CO lines are sensitive to non-thermal gas excitation, such as heating by X-rays and cosmic rays or turbulence, which might be significant in intensely star-forming DSFGs. Indeed, recent studies point towards highly excited CO rotational lines in strongly lensed DSFGs [35, 43]. Depending on the complexity of the data, the models can range from static, 1-D gas slabs [44–47] to fully 3-D models [48] and might incorporate time evolution.

deshima’s octave-wide bandwidth will allow simultaneous observations of multiple emission lines in the bright DSFGs. Such multi-line spectroscopy is critical for constraining different physical properties. However, previous studies with wide-band spectrometers such as Z-Spec were limited to a handful of very bright sources [49–51]; deshima 2.0 will expand this approach to a much larger sample of DSFGs. In particular, with the exception of the brightest *Planck*-selected sources, the high-excitation CO emission in DSFGs remains almost completely unexplored. deshima 2.0 should detect the high-*J* CO lines in the bright *Planck* and SPT DSFGs in only a few hours on-source.

As a demonstration, Fig. 3 shows a simulated spectrum of J1329+ 2243, the most FIR-luminous source at $z\ge 3$ from the samples considered in Fig. 2, with extensive archival CO observations [35]. We adopt a nominal chip design with 347 frequency channels spanning the 220–440 GHz range (*R* = 500). The response function of individual filters follows a Lorentzian profile with a peak coupling efficiency of 13.6% (based on the laboratory tests of the deshima 2.0 chip) We assume PWV = 0.6 mm, source elevation of 40 degrees, and a total on-source time of 3 hr. Even with such a short integration, we expect robust detections of multiple CO lines. Moreover, we will cover the potentially bright H$_2$O and H$_2$O$^+$ lines.

Finally, we note that due to the relatively low spectral resolution (R$\sim$500), several emission lines might blend together: particularly CO(7–6) and [CI](2–1) (rest-frame frequency separation $\Delta f_0=2.69$ GHz, $\Delta v \sim$ 1000 km/s) and CO(9–8) and OH$^+$ ($\Delta f_0=3.86$ GHz, $\Delta v \sim$ 1100 km/s). The latter pair is particularly susceptible to blending as OH$^+$ often traces the out-/in-flowing gas and might be seen in absorption [43, 52]; consequently, the CO(9–8) flux measured from $R\sim 500$ spectra might be significantly over/underestimated.

## Conclusions

We have presented the high-redshift extragalactic science case for the deshima 2.0 integrated superconducting spectrometer, which will be mounted at the 10-m ASTE telescope in 2022. Thanks to its combination of an octave-wide bandwidth, access to high frequencies and competitive sensitivity, deshima 2.0 will allow science-grade observations of high-redshift galaxies. The first integrated chip has been manufactured and tested in the lab. In terms of sensitivity, deshima 2.0 is already competitive with the Z-Spec grating spectrometer, and might become competitive with APEX.

The upcoming Science Verification Campaign will: (1) measure redshifts for $\sim$30 *Herschel*-selected galaxies at $z\sim 1$ and $z\ge 4$; (2) obtain multi-line spectroscopy of 5–10 strongly lensed DSFGs to study the physical conditions in these extreme sources. These figures are conservative; with further sensitivity improvements, the campaign can be expanded significantly. These deshima 2.0 observations will pave the way for future large-scale spectroscopic campaigns with ultra-wideband, multi-pixel MKID spectrometers [53] on, e.g., the planned 50-m AtLAST telescope [54] which will determine redshifts and physical properties of thousands of DSFGs.

## Footnotes

1 For a detailed summary, see the contribution by Taniguchi et al., in this volume [21].

2 nFLASH230, SEPIA345, nFLASH460. The sensitivity calculations are based on https://www.apex-telescope.org/ heterodyne/calculator/ns/index.php.
